# Clinical detection of deletion structural variants in whole-genome sequences

**DOI:** 10.1038/npjgenmed.2016.26

**Published:** 2016-08-03

**Authors:** Aaron C Noll, Neil A Miller, Laurie D Smith, Byunggil Yoo, Stephanie Fiedler, Linda D Cooley, Laurel K Willig, Josh E Petrikin, Julie Cakici, John Lesko, Angela Newton, Kali Detherage, Isabelle Thiffault, Carol J Saunders, Emily G Farrow, Stephen F Kingsmore

**Affiliations:** 1 Center for Pediatric Genomic Medicine, Children’s Mercy Kansas City, Kansas City, MO, USA; 2 Heartland Institute for Clinical and Translational Research, University of Kansas Medical Center, Kansas City, KS, USA; 3 Department of Pediatrics, Children’s Mercy Kansas City, Kansas City, MO, USA; 4 Department of Pediatrics, University of Missouri-Kansas City, Kansas City, MO, USA; 5 Department of Pathology, Children’s Mercy Kansas City, Kansas City, MO, USA; 6 Rady Children’s Institute for Genomic Medicine, San Diego, CA, USA

## Abstract

Optimal management of acutely ill infants with monogenetic diseases requires rapid identification of causative haplotypes. Whole-genome sequencing (WGS) has been shown to identify pathogenic nucleotide variants in such infants. Deletion structural variants (DSVs, >50 nt) are implicated in many genetic diseases, and tools have been designed to identify DSVs using short-read WGS. Optimisation and integration of these tools into a WGS pipeline could improve diagnostic sensitivity and specificity of WGS. In addition, it may improve turnaround time when compared with current CNV assays, enhancing utility in acute settings. Here we describe DSV detection methods for use in WGS for rapid diagnosis in acutely ill infants: SKALD (Screening Konsensus and Annotation of Large Deletions) combines calls from two tools (Breakdancer and GenomeStrip) with calibrated filters and clinical interpretation rules. In four WGS runs, the average analytic precision (positive predictive value) of SKALD was 78%, and recall (sensitivity) was 27%, when compared with validated reference DSV calls. When retrospectively applied to a cohort of 36 families with acutely ill infants SKALD identified causative DSVs in two. The first was heterozygous deletion of exons 1–3 of *MMP21 in trans* with a heterozygous frame-shift deletion in two siblings with transposition of the great arteries and heterotaxy. In a newborn female with dysmorphic features, ventricular septal defect and persistent pulmonary hypertension, SKALD identified the breakpoints of a heterozygous, *de novo* 1p36.32p36.13 deletion. In summary, consensus DSV calling, implemented in an 8-h computational pipeline with parameterised filtering, has the potential to increase the diagnostic yield of WGS in acutely ill neonates and discover novel disease genes.

## Introduction

Mendelian diseases, *in toto*, consume substantial healthcare resources.^1–3^ Recent advances in genomics technologies and computational analysis have yielded unprecedented progress towards understanding the relationship of genomic variation to infant morbidity and mortality.^4^ Genetic diseases, chromosomal aberrations and congenital malformations are the leading cause of infant mortality in the US.^5–7^ By detecting disease causing single nucleotide (nt) variants (SNVs) and small nucleotide (<50 nt) insertions and deletions (indels), rapid whole-genome sequencing (WGS) has accelerated and improved the sensitivity for the diagnosis of genetic illness in neonates.^8–10^ We have recently shown that over one half of acutely ill newborns with likely genetic diseases, enroled from a neonatal intensive care unit (NICU), were diagnosed using rapid WGS, with a median time of 23 days between consent and reporting of results.^9,10^ However, disease-causative alleles are not always nucleotide variants. Structural variants (SVs, copy number variations, translocations and inversions >500 bp in length) also contribute prominently to birth defects, Mendelian diseases and complex genetic diseases.^11^

Insertion and deletion copy number variants (CNV) are the most common types of SV.^12^ Although current estimates are subject to the limitations of identification by current technologies, the average diploid human genome differs from the reference genome by at least 700 CNVs, totalling at least 11 Mb,^13,14^ and encompassing ~400 genes.^13,15–19^ *De novo* CNV mutations arise via genomic rearrangements, *in cis* (via intra-chromosomal events) or *in trans* (interchromosomal), and through nonallelic homologous recombination and nonhomologous recombination. CNV length varies from a few hundred nucleotides to tens of millions.^20,21^ Their size distribution is skewed, with smaller SVs being the most frequent.^15,22^ CNV rates vary widely at different loci (1.7×10^−6^ to 1×10^−4^ per locus per generation).^23^ The mutation mechanism and selection pressure differ between insertion and deletion SVs (DSV).^22^ DSVs are approximately three times more common than insertions, and meiotic DSV rates in human sperm at four hotspots were shown to be at least twofold higher than for insertions.^24,25^ There is a strong purifying selection for deletions in exons and introns due to their potential for deleterious phenotypes.^22^ Thus, the impact of DSVs can range from having no discernible outcome to being incompatible with life.^12^

DSVs are known to be the most common type of mutation for many single gene diseases, including Duchenne Muscular Dystrophy, juvenile Batten disease, Spinal Muscular Atrophy, Pelizaeus–Merzbacher Disease, Williams–Beuren syndrome, Smith Magenis syndrome, Hereditary Neuropathy with Liability to Pressure Palsy, Miller–Dieker lissencephaly, 22q11.2 deletion syndrome, Thalassaemia and Ichthyosis, among many others.^12,15^ In other conditions such as Neurofibromatosis type 1, Tuberous Sclerosis, Sotos syndrome, CHARGE syndrome, Gaucher disease, Pituitary dwarfism and red green colour blindness deletions are less common^12,15^ but an important source of gene disruption justifying the need for comprehensive deletion detection. *De novo* DSVs are a risk factor for autism spectrum disorder^26^ and rare DSVs have been associated with schizophrenia.^27^ DSVs near genes are also thought to contribute to Crohn's disease, psoriasis and osteoporosis.^22^ While specific genetic tests are available for molecular diagnosis of many of these disorders, an as yet unattained goal for clinical WGS is to include the identification of a broad spectrum of DSVs for comprehensive aetiologic diagnosis of genetic diseases.

Karyotyping and fluorescence *in situ* hybridisation (FISH) have historically been used to identify large SVs (>0.5 Mb), but are limited by poor resolution.^11^ Array comparative genomic hybridisation (array CGH) and single nucleotide polymorphism (SNP) arrays are the current gold standard methods for detection of disease-associated CNVs.^13^ Array CGH involves comparative hybridisation of a test and reference sample, with inference of CNV gain or loss from signal ratio. For SNP arrays, a single sample is hybridised, and SNP probe log ratios are used to detect CNV gains or losses.^28^ These methods have lower breakpoint resolution than sequencing, and balanced structural rearrangements, e.g., translocations, inversions, are not detectable. Unless custom designed for specific loci, these methods do not generally detect SVs<10 kb.^29^

Short-read WGS data can also be used to detect insertion and DSVs.^29^ Paired end mapping (PEM), depth of coverage (DOC), split read mapping (SRM) and local assembly are the four principal methods used to detect SVs in WGS data.^30^ The DOC approach identifies CNVs by read depth in sequential genomic windows that is greater or less than a predefined (e.g., using a parametric model) or dynamically determined background level. Paired ends are nucleotide sequences from the ends of DNA fragments. In PEM, pairs with a mapping distance congruent with the intended DNA fragment size and expected orientation are deemed concordant. Non-concordant pair mapping signatures are used to infer if an event is an insertion (mapping distance less than expected), deletion (mapping distance greater than expected), inversion (mapping orientation opposite to expected) or translocation (pairs map to different chromosomes). In PEM, the maximum detectable insertion size is limited by the library fragment length, but there are no size limits for detection of DSVs or translocations. The SRM method identifies CNV breakpoints occurring within reads. In SRM, at least one of the segments resulting from read bifurcation must align to a unique genome location. Of these four modes of SV discovery, whole-genome assembly may hold the greatest, long-term promise for accurately typing all SV forms.^15^ Unfortunately none of these approaches is comprehensive. In the interim, for a typical WGS sample, large proportions of validated SVs will be unique to each method. Although only DOC accurately predicts absolute gains or losses, it does not resolve breakpoints well. PEM requires consistent fragment sizes and performs poorly in repetitive loci. Similarly, SRM and short-read sequence assembly are unreliable in non-unique regions.^29^ Numerous permutations of these four paradigms have been implemented as computational tools, yet there is a lack of consensus regarding which have the best performance and standard SV detection pipelines for use in WGS for the diagnosis of genetic diseases does not yet exist.

Clinical grade SV detection is critical both for the diagnosis of certain genetic disorders, and, broadly, for the assessment of missing causative haplotypes. Here we report comparisons of several existing tools for detection of SVs in WGS data, and the development of an improved SV detection pipeline—SKALD (Screening Konsensus and Annotation of Large Deletions)—based on consensus, filtered SV calls. We focused initially on DSVs since they are the most numerous,^18,24,31^ deleterious^25^ and readily detectable type of SV in paired end WGS data.^30^

We also report an initial application of SKALD for molecular diagnosis of genetic diseases by rapid WGS of familial trios or quartets in which the proband was an acutely ill infant receiving care in an intensive care unit. Specifically, we demonstrate how the integration of SKALD DSV detection into a WGS variant detection pipeline might provide a more comprehensive molecular diagnosis strategy for genetic disease in a time-frame consistent with clinical management decisions.

## Results

### Evaluation of structural variation detection tools using simulated WGS data

A literature survey identified 50 software tools (Supplementary Table S1) capable of detecting SVs in short-read WGS. The methods used a variety of approaches to detect SVs (Table 1): DOC methods detected DSVs on the basis of a local decrease in mapped read depth compared with unaffected, flanking regions. PEM methods predicted DSVs on the basis of significantly increased interval between the coordinates of mapped read pairs relative to those mapping to unaffected, flanking regions.^30^ Ten of the 50 methods did not require substantial effort for installation or execution, did not require a control sample, supported the widely used .bam format,^32^ and could be run concurrently on multiple processors (Table 1). The performance of these 10 methods in DSV detection was evaluated using a simple human chromosome 1 DSV simulation set that featured perfect read matches (no nucleotide variants or sequence errors) and 200 homozygous DSVs of size 500–10,000 nt. Read depth, repetitive regions and GC content can influence the accuracy of DSV predictions.^33^ Comparison of these attributes between the simulated set and Chr 1 reference data showed that GC content and repeat feature frequency differed by <10%, and the simulation mean read depth was found to be nearly identical to the target ×40 (Supplementary Figure S1). Simulated DSVs had stretched read pairs spanning breakpoints with uniform inner and outer read depths, required for PEM and DOC detection methods, respectively (Supplementary Figures S2 and S3).

Six methods identified true positive (TP), simulated DSVs in this simulation (Breakdancer (BD), Clever, Control-Freec, ERDS, GenomeStrip (GS) and Lumpy; Table 2). GS, BD and ERDS exhibited the best performance, with recall (sensitivity or TP rate) of 54%, 37% and 55%, respectively, and precision (positive predictive value (PPV)) of 18%, 81% and 11%, respectively (Table 2). However, these values were less impressive in light of the simplicity of the test data set and requirement for calls to overlap a reference DSV by only 1 nt to be classified as TP. In contrast, the sensitivity and recall of nucleotide variant calls in clinical WGS with best practice methods are >99.5% and >99.9%, respectively.^9,34^

**Figure 1:**
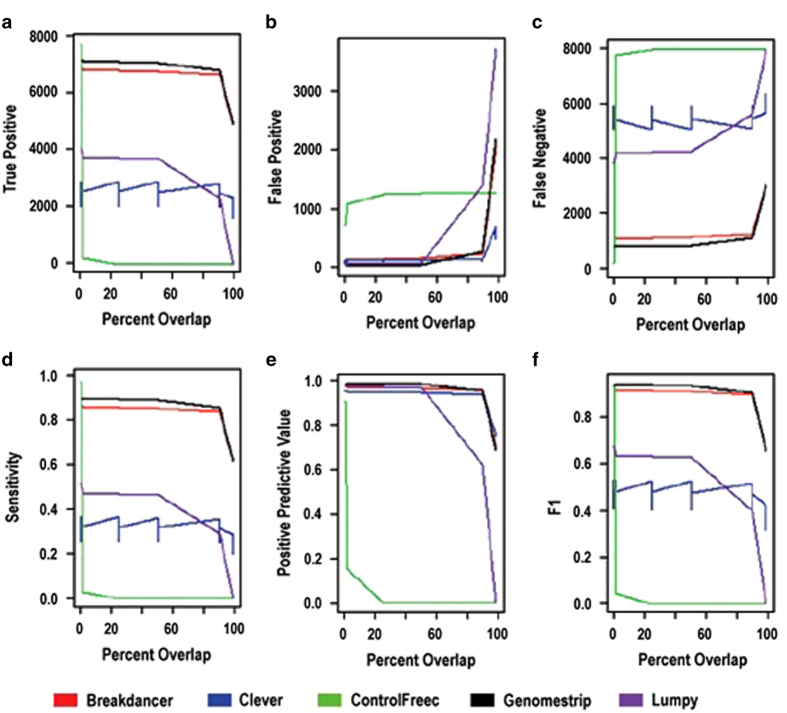
Performance of five DSV detection tools as determined by reciprocal overlap of predictions from three iterations on one of three WGS simulations. Shown are true positives (TP, **a**), false positives (**b**), false negatives (**c**), sensitivity (recall, **d**), positive predictive value (precision, **e**) and F2 measure (**f**). ERDS did not yield any TP DSVs in this simulation. Similar results were observed for two other WGS simulations (data not shown).

Performance of the six methods that identified TP, simulated DSVs was further evaluated using a more complex, genome-wide DSV simulation set that included homozygous and heterozygous DSVs, and typical WGS rates for nucleotide variants and sequencing errors. In the latter, BD and GS exhibited superior recall (or sensitivity), precision (PPV) and F2 measure (Figure 1). The difference in sensitivity between homozygous and heterozygous deletion predictions of these methods was <1%.

In contrast to nucleotide variant identification, the overlap of the start and end coordinates of predicted and actual DSV were imprecise. Thus, the performance of the methods decreased as the required overlap between predicted and actual DSV coordinates increased. BD and GS alone, however, had stable performance metrics between overlap of predicted and actual DSV coordinates between 1 nt and 87% (Figure 1). In contrast, e.g., the performance of Lumpy dropped substantially at an overlap requirement >50%. The sensitivity (recall) of BD and GS at 87% overlap of predicted and actual DSV coordinates was 85% and 88%, respectively (Figure 1). The precision (PPV) of BD and GS at the same overlap were 93% and 92%, respectively.

Mechanistically, the results of the WGS simulation implied that PEM (BD) was the single best method for DSV detection, albeit a combination of PEM, DOC and SRM methods (GS) was optimal. We explored how to combine the results of BD and GS to achieve highest analytic performance. Compared with the BD or GS alone, consensus DSV calls from a combination of the two methods with 90% prediction overlap (BD ∩^90%^ GS) was 3.8% less sensitive than BD or GS alone, and yielded a 3.3% improvement in precision (PPV). Given the lack of net improvement in analytic performance of BD ∩^90%^ GS, we elected to seek maximal sensitivity (recall) by combining the DSV calls from BD and GS (BD U GS), and then to identify and apply filtering steps that would confer high precision (PPV).

### Evaluation of BD and GS with four experimental WGS replicates of NA12878

NA12878, a HapMap CEU trio proband^35^ was chosen for experimental analysis of DSV detection methods, as it had been extensively sequenced by the 1,000 genomes project (1KGP),^36^ and has been selected as SV benchmark by the National Institutes of Standards and Technology.^37^ We generated four experimental NA12878 data sets comprising two 2×250 nt and two 2×100 nt 40× WGS.

### Establishment of classifier for improved precision of DSV predictions in combined BD and GS calls

We sought out to establish a classification algorithm to maximise precision (PPV) in combined DSV calls from BD and GS (BD U GS). Three different classification algorithms were evaluated and model attributes were either those used in the DOC and PEM methods or derived from visualisation of TP and false positives (FP) DSV predictions. For example, when viewed in Integrated Genome Viewer (IGV, Broad Institute, Boston, MA, USA), many FP DSVs were very large or were associated with ineffective unique read mapping to repetitive regions. The initial set of DSV precision attributes chosen for parameterisation in the classification algorithms were: Maximum depth ratio (ratio of read depth within a DSV to that flanking the DSV), DSV size, number of supporting paired reads, number of repeat features that overlap the DSV call and number of exons overlapping the DSV call.

**Figure 2:**
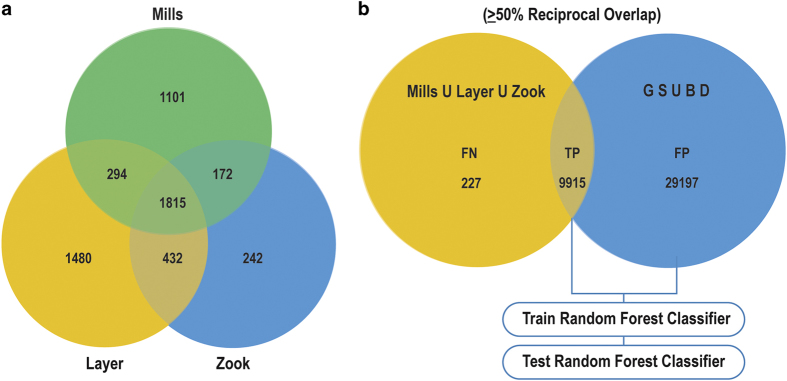
Development of a DSV prediction classifier with reference data. (**a**) Concordance of confirmed DSVs among three published NA12878 reference DSV sets (Mills *et al.*,^40^ Layer *et al.*^41^and Zook *et al.*^42^). DSVs were considered concordant if their chromosomal coordinates had a reciprocal overlap of at least 50%. About 33% of DSV calls were common to all three sets; 49% were common to more than two sets. Rates of concordance were lower if the overlap requirement was increased. (**b**) Process employed to develop a DSV prediction classifier. FN, false negative; FP, false positive; TP, true positive. Numbers shown represent a 50% reciprocal intersection between the union of DSVs from Mills *et al.,*^13^ Layer *et al.*^41^ and Zook *et al.*,^42^ and the union of GS and BD calls for NA12878 technical replicates. TPs were defined as calls which had a ⩾50% reciprocal overlap between Mills U Layer U Zook and GS U BD. FNs were defined as Mills_Layer_Zook DSVs that were not found to have a ⩾50% reciprocal overlap with GS U BD calls. FPs were defined as GS U BD calls not found to have a 50% reciprocal overlap with Mills_Layer_Zook DSVs.

Linear discriminate analysis, logistic regression and random forest are three common algorithms for establishing a classification model.^38,39^ There is currently no all-encompassing gold standard set of DSVs for any human genome. HapMap sample NA12878, from a US female of northern European ancestry, has become the gold standard for nucleotide variant calls. During this study, three independent sets of DSV coordinates from NA12878 were published that had been verified by more than one technology (Mills *et al.*,^40^ Layer *et al.*^41^ and Zook *et al.*,^42^ respectively; Figure 2a), containing 3,382, 4,021, and 2,664 DSV calls, respectively. With the caveat that DSV call concordance is highly dependent on the cutoff in overlap of chromosomal start and stop coordinates, when requiring a reciprocal overlap in chromosomal coordinates of >50%, 1,815 (33%) of DSVs were common to the 3 reference sets. Thus, while extremely useful, none of these alone represented a complete gold standard set. In total, they contained 5,536 unique DSVs (with <50% overlap in chromosomal coordinates). Likewise, chromosomal microarrays (SNP arrays) are considered the gold standard for clinical diagnostic testing of DSVs. We generated two Affymetrix SNP array (Thermo Fisher Scientific Inc., Santa Clara, CA, USA) data sets for sample NA12878. They contained only 2% (131) and 3% (175) as many DSV calls as the verified sets. Furthermore, only 67 (28%) of DSVs were common to the two SNP array sets (when requiring a reciprocal overlap in chromosomal coordinates of >90%). The average size of DSV detected by SNP array was ~10,000 nt, compared with ~1,000 nt with BD and GS (Supplementary Figure S4). Thus, SNP arrays are insensitive for detection of DSVs, particularly DSVs of size <10,000 nt. In light of these results, SNP array-based DSV calls were considered to lack sufficient sensitivity or precision for refinement of GS and BD DSV performance.

We generated four replicate WGS data sets from NA12878, obtained BD and GS DSV predictions for each, and created Training and Test DSV data sets from the superset of BD and GS DSV predictions from the four NA12878 WGS replicates (BD U GS). DSV predictions in individual experimental replicates varied from 4,641 to 22,080, partly reflecting differences in sequencing instrument and read length. The reference set comprised the superset of the Mills *et al.*, Layer *et al.* and Zook *et al.* DSV calls. Any NA12878 replicate DSV prediction which had a reciprocal overlap in chromosomal coordinates with a reference DSV of <50% was considered TP. DSV predictions not meeting this criterion were considered FP (Figure 2b).

Of the three classification algorithms examined, the random forest method had the best analytic performance, when trained with classified DSVs from all four NA12878 WGS replicates and tested on all four NA12878 technical replicates. On average, the random forest classifier improved precision (PPV, TP/(TP+FP)) from 0.35 to 0.78, while decreasing recall (sensitivity, TP rate, TP/(TP+FN)) from 0.32 to 0.27. Thus, on average, the random forest classifier improved the F2 measure (from 0.32 to 0.52, Table 3).

### Analysis of precision in replicate sets before and after filtering

The run-to-run precision of DSV predictions was assessed in two samples (U173 and pg96), each with three WGS replicates (r1, r2 and r3). Despite material differences in the WGS methods used in the replicates, 89.8% and 27.7% of DSV calls were common to at least two of three replicates (U173 and pg96, respectively). Given the methodological differences in WGS runs, we expected greater similarity for r2∩^50%^r3 than for r3∩^50%^r1 or r2∩^50%^r1, which matched actual results (Table 4). Application of the classification tool decreased the number of DSV predictions by 2.8-fold and 4.1-fold in U173 and pg96, respectively (Table 4). In contrast, it decreased the r1∩^50%^r2∩^50%^r3 class by only 2.5-fold and 2.4-fold, respectively (Table 4). Following filtering, 96.1% and 52.4% of calls were common to at least two replicates, respectively.

**Figure 3:**
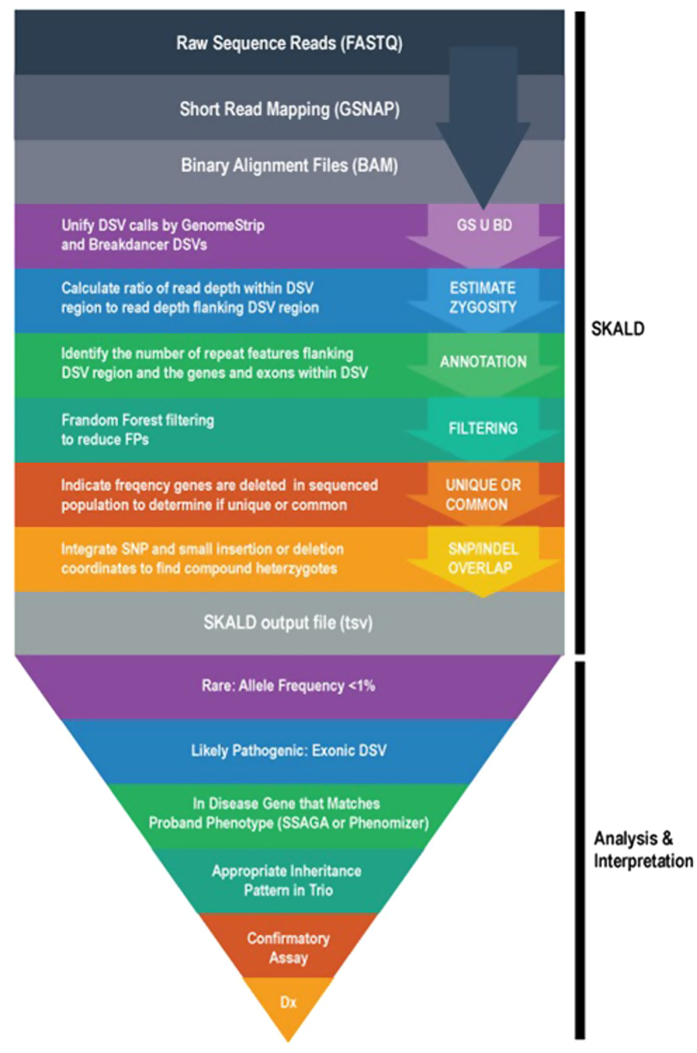
A flow diagram of the SKALD pipeline and downstream analysis for detection of likely disease-causative DSVs in WGS. After reads were aligned, GS and BD were executed concurrently on bam files from parent–child trios. Filter attributes, overlap % and annotations were obtained for each BD U GS DSV prediction. Since genes that were commonly deleted were unlikely to be deleterious, the population frequency of DSVs overlapping genes helped determine whether the DSV was likely to cause a rare genetic disease. Finally to identify likely pathogenic compound heterozygote states, any SNVs or indels overlapping a DSV were included as part of the SKALD output in the form of a tab separated text file.

The random forest classifier, and BD U GS DSV calls were incorporated into a computational pipeline called SKALD (Figure 3). SKALD included DSV attributes, such as size, zygosity and degree of overlap between BD and GS predictions, and annotations, such as exon content, repetitive element content, OMIM disease association and population frequency. SKALD was designed to run in parallel with nucleotide variant calling and genotyping algorithms on test subjects with diseases of unknown but likely genetic aetiology. Computation completed on most WGS samples within 8 h.

### Identification of disease-causative DSVs in WGS of acutely ill neonates

The diagnostic utility of SKALD was examined in 36 families with an acutely ill infant suspected of having a genetic disorder.^9,10^ Fourteen families were evaluated by WGS of singleton, affected probands, 1 family comprised WGS of a mother–infant proband duo, 20 families were parent–infant trios and 1 family was analysed by WGS of a quartet (2 affected infants and both parents). One sample from an unrelated, unaffected individual (NA12878) was used as the control in trio and quartet comparisons. Sample descriptions and WGS run quality metrics are available in Supplementary Tables S2 and S3. Human genome GRCh37.p5 was used as the reference version for alignments and simulations. For most samples, WGS was with 2×100 nt reads, with a fragment size of 200–400 nt, and mean read depth was 34.8+6.0-fold coverage. Five DSVs, detected by SKALD in five probands, were chosen at random and assessed for validity in the respective trios by quantitative PCR (qPCR; Supplementary Table S7). Thirteen of 15 DSVs were TPs, yielding a PPV of 87%. DSVs which overlapped genes considered to be causative for undiagnosed probands were identified by SKALD in 2 of 36 families, yielding an incremental diagnostic rate of 6%.

### Case 1—two siblings with heterotaxy

CMH184 was a 6-week-old male with visceral heterotaxy and congenital heart disease (dextro-transposition of the great arteries with total anomalous pulmonary venous return) enroled from the Children’s Mercy hospital (CMH) NICU. A 6-year-old brother (CMH185) had nearly identical findings. Parents (mother CMH186 and father CMH202) and two other siblings (one male and one female) were healthy. Among 8,050 and 7,280 DSVs identified by SKALD in CMH184 and CMH185, respectively, was a 5,904 bp heterozygous DSV of *MMP21* exons 1–3 (chr10:127460915–127466819), that was present in CMH184, CMH185 and the unaffected mother CMH186 (Supplementary Tables S4 and S5). The nucleotide variant calling pipeline identified an apparently homozygous single nucleotide deletion that induced a frameshift in *MMP21* for CMH184, CMH185 and the unaffected father (CMH202) (c.365del (p.Met122SerfsTer55); Supplementary Figure S5). Familial relationships were confirmed by segregation analysis of private variants, suggesting a deletion of the maternal allele. The *MMP21* frame-shift variant was confirmed by Sanger sequencing in CMH184, CMH185 and CMH202, and absent in the unaffected mother CMH186. The *MMP21* heterozygous large deletion encompassing exon 1–3, was validated by SNP microarray analysis in the affected proband, and breakpoints were identified by long-range PCR and Sanger sequencing in the trio. The proband and his brother are now 3 and 9 years old, respectively, and in relatively good health following corrective cardiac surgeries. *MMP21* encodes matrix metalloproteinase 21, which is involved in breakdown of the extracellular matrix during embryonic development.^43^ Zebrafish and mice lacking MMP21 exhibit heterotaxy and transposition of the great arteries,^44^ similar to that observed in CMH184 and 185.

### Case 2—a newborn with dysmorphic features and cardiac defects

CMH773 was a 6-day-old full-term newborn female with dysmorphic features, a ventricular septal defect and persistent pulmonary hypertension. Parents CMH774 and CMH775 were unaffected. The pregnancy was complicated by IUGR and polyhydramnios, although Apgar scores at birth were good (8 at 1 min, and 9 at 5 min). The proband was at the 8th percentile for weight, 35th percentile for length and 27th percentile for occipito-frontal circumference. She had a prominent forehead, redundant nuchal folds, low and wide spaced hypoplastic nipples, high arched palate, a low set thumb and a prominent nasal bridge. She had a spontaneous pneumothorax and was ventilator dependent. FISH analysis for Turner syndrome and Trisomy 21 were negative. A 12 gene next generation sequence analysis for Noonan syndrome was negative. A 13.6 Mb heterozygous Chr 1p36.32p36.13 DSV, consistent with proximal 1p36 deletion syndrome,^45–47^ was found by SNP microarray and confirmed by FISH (chr1:4,848,728–18,503,068del). Among 7,855 DSVs, SKALD retrospectively identified the same mutation (Supplementary Table S6). This DSV was associated with monosomy for at least 170 genes. Chr 1p36 microdeletion syndrome is associated with two distinct syndromes: classic distal 1p36 monosomy syndrome features a DSV of the distal terminal 6 Mb.^48^ Proximal 1p36 deletion has variable size and clinical features, including poor prenatal and postnatal growth, seizures, developmental delay, hirsutism, cardiovascular malformations, microcephaly, limb anomalies and craniofacial dysmorphology, including frontal and parietal bossing.^45–49^ The distal breakpoint in proximal 1p36 deletions can overlap with the classic 1p36 deletion, while the proximal breakpoint can extend to 1p36.1. A 16 Mb 1p36 DSV that covered both the distal and proximal deletions was reported in an infant who died neonatally.^49^ Chromosome analysis of the trio showed the 1p36.32p36.13 deletion to be *de novo* in CMH773 (Supplementary Figure S6). During WGS and microarray analysis, the infant’s condition deteriorated. Her parents elected palliative care, and she expired on day of life 10. SKALD was retrospectively applied to this case and identified the same DSV. The clinical microarray took 21 days to return results. If SKALD had been available at the time of enrolment, the mutation may have been identified 10 days earlier, potentially prior to her death.

## Discussion

If outcomes are to be improved for acutely ill neonates suspected of having a genetic disorder, it is crucial to confirm or refute a molecular diagnosis in a timely manner. The potential of WGS to inform healthcare providers in this regard has not yet been fully realized. In particular, comprehensive, clinical SV identification methods for paired short-read WGS are needed. This is particularly true for exonic DSVs for which likely pathogenicity and causality for clinical features in affected patients can often be readily ascertained. The challenge of DSV identification with paired, short-read WGS is more analytical than technical.^15^ While many DSV detection tools have been developed, the absence of robust truth sets or benchmarking studies has been a significant impediment to progress. Thus, there is not yet a default ‘gold standard’ commonly employed programme, as currently exists for WGS nucleotide variant detection (i.e., GATK^50^). Herein we report results of benchmarking studies of 10 DSV detection tools, using WGS DSV simulation sets with certain characteristics based on a validated set of CNVs from 185 of the 1,000 genome samples. Analyses of analytical performance of available SV detection tools in simulation sets led to the nomination of BD and GS as best. BD and GS had reasonable analytic sensitivity (recall, 85% and 88% at 87% overlap of predicted and reference DSV coordinates, respectively) and specificity (PPV, 93% and 92%, respectively) in a WGS DSV simulation set. Performance, however, degraded rapidly if the required overlap between predicted and reference DSV coordinates was >87%. Thus, these tools do not typically yield start and stop coordinates with nucleotide precision. Furthermore, since, the simulation set did not reproduce all of the types of noise or imprecision encountered in experimental WGS data, we sought to combine the methods to achieve more robust performance.

The best combination of the BD and GS methods was empirically determined to be the union of all BD and GS DSV calls (BD U GS), followed by filtering with a random forest-based classifier. The classifier was trained with three sets of validated DSVs from the reference sample NA12878, and four NA12878 paired, short-read experimental WGS replicates. The resultant precision (PPV) was 78%, recall (sensitivity) was 27% and the F2 score was 52%. While these values were less than those achieved by nucleotide variant calling tools in WGS, it should be noted that only 33% of DSV calls in the three validated DSV sets used for training were common to all three. Thus, considerable further work will be needed before there exists comprehensive, validated sets of SVs for reference samples with which to undertake additional training. The hybrid BD U GS and classification method was incorporated into a computational pipeline, together with annotation tools to facilitate interpretation with regard to likely pathogenicity for genetic diseases. This resultant pipeline was called SKALD.

For diagnosis in acutely ill neonates, time to result is another key parameter that has not hitherto been benchmarked for DSV detection tools. For routine clinical use, DSV detection should be performed in parallel with nucleotide variant calling, with a similar time to result. Ideally, resultant nucleotide vcf and DSV call files would be amalgamated, annotated and analysed together. SKALD currently has a turnaround time of <8 h per WGS data set.

The approach whereby SKALD was used to aid in diagnosis of DSV-associated disorders in newborns herein primarily utilised familial trio WGS. SKALD DSV calls in the proband underwent a set of filtering steps: First, DSVs that did not contain genes or only containing commonly deleted genes were removed.^51^ The latter was a powerful filter, since rare, highly penetrant genetic diseases cannot be causally associated with common DSVs.^8^ Second, SKALD DSV calls were filtered to retain genes causing known genetic diseases that shared clinical features with the proband’s specific phenotype^8^ (Figure 3). This was also a powerful filter, given the availability of tools such as Phenomizer and SSAGA to nominate disease genes comprehensively on the basis of clinical features. Phenomizer contains both Orphanet and OMIM disease entries, and thus has good representation of contiguous gene deletion syndromes, as well as single gene disorders. However, this filter had less utility when the proband’s disease features were atypical, reflective of an early phase in disease evolution, represented more than one condition or if the patient had a novel genetic disease. The combination of allele frequency and phenotypic filters allowed for greater tolerance of non-specificity, which, in turn, allowed greater analytic sensitivity. These filters also greatly accelerated analysis time. Third, parental WGS were inspected to determine whether the candidate DSV fit a recognised inheritance pattern. Finally, for recessive conditions with a heterozygous candidate DSV, the filtered SKALD DSV calls were combined with rare, potentially pathogenic nucleotide variants in the proband to identify heterozygous nucleotide variants *in trans*.

While analytic performance was imperfect, SKALD nevertheless had diagnostic utility among 36 families with acutely ill infant probands with likely genetic diseases. SKALD identified or confirmed two genetic diagnoses: In the first family, with *MMP21*-associated heterotaxy, a diagnosis would not have been made without the dual use of SKALD and a nucleotide variant detection pipeline. The affected siblings had compound heterozygosity for a pathogenic nucleotide variant and a small (5,904 nt), exon-deleting DSV. This family was also remarkable since *MMP21* had not previously been associated with heterotaxy in human. In the remaining infants, SKALD recapitulated the diagnosis of a large DSV that was made by cytogenetic analysis and SNP array. SKALD was retrospectively applied in this case, but had it been performed in parallel with nucleotide variant analysis the diagnosis may have been made sooner. These cases clearly indicate the potential utility of DSV detection as part of WGS for diagnosis of genetic diseases.

In light of this experience, it is interesting to consider what near-term role SKALD might play in the diagnostic work-up of likely genetic diseases. First, trio WGS is expensive, and the only current application where it is likely to be cost-effective is for diagnosis in acutely ill patients in whom a genetic disease is likely. In such patients, the primary subjects of analysis and interpretation are nucleotide variants. In this situation, DSVs constitute a second set of potentially primary findings that can be obtained at small incremental cost. The latter includes cost of interpretation and confirmatory assays—either PCR with Sanger sequencing of breakpoints or SNP/CNV array support—prior to reporting of results. Thus, SKALD and nucleotide variant calling have unique potential to diagnose genetic conditions with causal compound heterozygous nucleotide variants and DSVs which hitherto were underdiagnosed. Clearly the breadth of use of WGS in genomic medicine, and thereby SKALD, will increase as cost effectiveness starts to be demonstrated.

It is also interesting to speculate what the near-term role of SKALD might be relative to ‘gold standard’ clinical testing for DSVs, which include array comparative genetic hybridisation, high resolution cytogenetic analysis, FISH, SNP/CNV arrays and exon arrays. Clearly SKALD is not sufficiently mature to be used as a stand-alone diagnostic test, even if clinically validated and performed in a CLIA/CAP compliant manner. However, SKALD has two relatively unique capabilities: First, it can detect small DSVs (hundreds to thousands of nucleotides) relatively comprehensively, while detection of a SV by array is dependent on probe density and placement. Second, SKALD can identify DSV breakpoints with higher precision than array at the nucleotide level, which could be important if the array has insufficient resolution to determine whether a structural variation affects a critical gene. In short, SKALD and array for DSVs appear to be highly complementary.

There are several limitations to the current study and tool. First, there exists considerable community need for comprehensive, validated sets of SVs in widely available reference genomes. While several recent manuscripts have described validated DSV calls for sample NA12878; the lack of concordance between these sets indicates that they remain inadequate for assessments of analytic performance. Without such, further training of classification tools is limited. Related to this is the need for large databases that provide population frequencies for DSVs in various ethnic groups, which will improve the performance of filtering common DSVs. Second, SKALD, as described herein, did not fully harness the power of SRM. This reflected the read alignment parameterisation and relatively short (100 nt) reads used herein. Optimisation of read alignment that is permissive to SRM and use of longer reads—such as 250 nt—are likely to improve the performance of the GS component of SKALD significantly. Finally, SKALD is currently limited to DSVs. Clearly the addition of copy number gains would be desirable for broadest utility.

In summary, the identification of DSVs by SKALD, when combined with nucleotide variant detection in WGS, appears to be effective for identifying genetic diseases in neonates. Having tested SKALD in a limited set of cases, we next propose to implement it in the larger Precision Perinatology 1 study (*PrePer1*, clinicaltrials.gov). *PrePer1* is a randomized, prospective study of the clinical utility and cost effectiveness of rapid whole-genome sequencing for genetic disease diagnosis and implementation of precision neonatology in a broader group of neonates in a level IV NICU setting. In this context it will be of great interest to quantify the incremental diagnostic yield of SKALD beyond that of nucleotide variants in WGS and conventional clinical tests for pathogenic SVs.

## Materials and methods

### Study participants

This study was approved by the Institutional Review Board of CMH. Informed written consent was obtained from adult subjects and parents of living children. DNA samples from 70 subjects were analysed. They were HapMap subject NA12878, obtained from the Coriell Institute for Medical Research, NJ, 2 CMH quality control samples, Pg96 and U173, and 36 families with an acutely ill infant suspected of having a genetic disorder who were enroled from the level IV NICU at CMH between November 2011 and October 2014.^8–10^ Fourteen families were evaluated by WGS of singleton, affected probands, 1 family comprised WGS of a mother–infant proband duo, 20 families were parent–infant trios and 1 family was analysed by WGS of a quartet (2 affected infants and both parents).

### Ascertainment of clinical features

The clinical features of NICU infants were ascertained comprehensively by physician and family interviews and review of the medical record. Baseline demographics including age, gender, gestational age, birth weight, APGAR scores and family history were collected. Phenotypic features were translated into Human Phenotype Ontology (HPO) terms and mapped to ~4,300 monogenic diseases with the clinicopathologic correlation tool Phenomizer.^9,10,52^ The HPO is developed using the medical literature, Orphanet, DECIPHER and OMIM. Briefly, Phenomizer assists in finding the correct clinical diagnosis by exploiting the semantic structure of the HPO. Phenomizer uses term-similarity measures to calculate a similarity score for query HPO terms entered by the user and terms used to annotate diseases in HPO. It then assigns a *P* value using statistical modelling to compare the similarity score obtained for the specific set of phenotypic terms entered to the distribution of similarity scores obtained using randomly chosen HPO term combinations. The *P* value was then used to rank the diseases.

### Whole-genome sequencing

Genomic DNA extraction from whole blood, library preparation, sequencing and data analysis were performed using validated protocols.^9,10^ Genomic DNA was prepared using Illumina TruSeq PCR Free sample preparation (Ilumina Inc., San Diego, CA, USA). Quantitation was by real-time PCR. Sequencing libraries had a fragment size of 200–400 nt.

For analysis of run-to-run precision of DSV predictions, WGS was performed three times in two samples (U173 and pg96, replicates r1, r2 and r3). The replicates were generated during methods development for clinical WGS, and utilised different Illumina sequencing instruments, sequencing-by-synthesis (SBS) chemistry and read lengths. Sample U173 WGS replicates were: r1. NextSeq500 instrument (Illumina) with 2×120 nt reads and version 4 (v4) SBS chemistry, r2. HiSeq 2500 instrument (Illumina) with 2×100 nt reads with v3 SBS chemistry and a 26 h recipe (rapid run mode) and r3. HiSeq 2500 2×100 nt reads with v4 chemistry and an 18 h recipe.^34^ Sample Pg96 WGS replicates were: r1. HiSeq 2500 2×250 nt reads, r2. HiSeq 2500 2×120 nt reads, 11 day protocol and v3 chemistry, and r3. HiSeq 2500 2×100 nt reads, v4 chemistry and 18 h recipe.^34^ All other samples underwent WGS once by 2×100 cycle SBS on Illumina HiSeq 2500 instruments in 26 h rapid run mode. WGS was to a minimum depth of 90 Gb per sample (Supplementary Tables S2 and S3), to provide an average 34-fold genome coverage. Each sample met established quality metrics.

Sequence data were generated with Illumina RTA 1.12.4.2 (Illumina) and aligned to the human reference GRCh37.p5 using GSNAP.^53^ Sequence analysis employed FASTQ files, the compressed binary version of the Sequence Alignment/Map format (bam, a representation of nucleotide sequence alignments). Analysis programmes were either written in Perl, R, Make or the Linux bashshell scripting language.

### Selection of DSV detection tools

The criteria for selection of DSV detection tools for evaluation from a total set of 50 tools surveyed (Supplementary Table S1) were: (1) Set-up and installation required only minimal if any third party tool or library dependencies (e.g., proprietary alignment tool) and did not require root access; (2) Execution was efficient and autonomous at the whole-genome level (e.g., could be successfully completed in clinically acceptable timeframe and did not require intermittent monitoring and restarting due to recurrent calibration or errors); (3) Execution did not require a control sample; (4) Supported the widely used .bam format; (5) Was still supported by the tool developers (e.g., had been updated in the last year, could receive response by e-mail from authors regarding questions); (5) Could be run concurrently on multiple processors; (6) Detected at least one Chr1 DSV, defined as >1 nt overlap in predicted coordinates, as part of a simulation test.

### WGS simulation data

For initial evaluation of the performance of SV detection tools, 270 homozygous DSVs of size 500–10,000 nt were created in a representation of human chromosome (Chr) 1 with 40× coverage and 2×100 nt paired reads. Reads were simulated from this modified Chr 1 GRCh37.p5 sequence file using wgsim 0.3.0^32^ (with default parameters). Simulated reads were aligned to the human reference GRCh37.p5 using GSNAP^53^ version 2012-07-12, and sam files were converted to the bam form using samtools^32^ 0.1.18. Overlaps of the genome coordinates of DSV predictions and those present in the simulated set were determined by standard Linux utilities and Bedtools 2.17.0.^54^ TP DSV calls were defined as DSV predictions that overlapped a simulated DSV by >1 nt.

Currently, there is no comprehensive ‘gold standard’ set of known DSVs for a reference WGS.^55,56^ The 1KGP has published a validated SV deletion set from 2,504 human genomes.^40,57^ To evaluate DSV identification tools at genome scale, three WGS samples with known deletions were simulated with parameters derived from phase 2 1KGP analyses.^40^ DSVs were simulated with random length (600–8,000 nt) and intra-chromosomal placement, while being distributed proportionally to chromosomes by their size at a rate of 1 per 400 Kb (~7,500 per sample). More recent phase 3 1KGP analyses demonstrated a slightly lower prevalence and median size of deletions than used in simulation data with ~2,800 deletions per sample having a median size of 2,455 nt for phase 3 1KGP published data compared with ~7,500 deletions per sample and median deletion size of 3,800 nt for our simulation data. Previous WGS experience was used to establish rates for SNPs (1 per Kb), small insertions and deletions (0.1 per Kb), and nucleotide errors (20 per Kb). Library insert size was 400 nt, read length was 2×100 nt and read depth was 40×. Read simulation, alignment and bam file creation were as before. Differences between WGS simulation and expected genome reference values for GC content, repetitive feature frequency and target depth were <10%, similar to the Chr 1 simulation (Supplementary Figure S1). Three independent WGS samples were simulated to reduce any potential tool deletion position advantages occurring by chance via random placement. To compare sensitivity for homozygous and heterozygous DSVs, the simulated DSVs were 98% heterozygous and 2% homozygous. A subset of DSVs was visually inspected for each sample using IGV (Supplementary Figures S2 and S3).

DSV tools were evaluated in three simulated samples. Each sample was evaluated three times to estimate precision. Tool predictions were compared with simulated DSVs (depicted as set notation where intersection is ∩, no intersection is $\bar{\cap }$, and union is U) at six discrete reciprocal overlap values (1 bp, 1, 25, 50, 90 and 99%) since, to our knowledge, no standard SV coordinate overlap criteria yet exist. Performance measures were TPs, FPs, false negatives (FNs), sensitivity (SENS), PPV and the F2 measure. With an unknown quantity of true negatives, the F2 measure substituted for specificity. TPs, FPs and FNs were counted and SENS, PPV and F2 were calculated for each tool (SENS=TP/(TP+FN); PPV=TP/(TP+FP); F2=(1+*β*^2^)×(SENS×PPV)/(*β*^2^×SENS+PPV) where *β*=2).

### Confirmatory testing for DSVs

Confirmatory testing for DSVs included long-range PCR, qPCR and Sanger sequencing of DSV breakpoints. For qPCR, DSV regions-of-interest were tested along with a separate normal locus that is used as an internal standard. Briefly, the ΔΔCt values were log converted and normalised to a reference gene (*OFD1*). NA12753 was used as a reference sample. Results from test samples were compared with the reference sample which contained two copies of the tested locus.

For SNP array analysis, isolated genomic DNA was prepared using a standard, eight-step Affymetrix Cytoscan assay (Thermo Fisher Scientific Inc.) protocol. Arrays were washed, stained and scanned. Raw .cel and .dat files were converted to .cychp files using Affymetrix CytoScan HD Array. Chromosome Analysis Suite 2.0 NetAffx 32.3 (hg19) was used for data analysis (Thermo Fisher Scientific Inc.) and export of DSV calls.

### Analysis and Interpretation of nucleotide and DSVs

Nucleotide variants were detected and genotyped with the Genome Analysis Toolkit (GATK) v. 1.4 or 1.6^31,50^ and yielded an average of 4.9 million nucleotide variants per sample (Supplementary Table S2). Variants were annotated with RUNES, noncommercial CMH software (Children's Mercy Hospital, Kansas City, MO, USA).^8,34^ WGS variant interpretations considered multiple sources of evidence, including variant attributes, the gene involved, inheritance pattern and clinical case history. Causative nucleotide variants were identified primarily with VIKING software^8,34^ by limitation to American College of Medical Genetics (ACMG) Categories 1–3 and allele frequency <1% from an internal database.^8–10,34^ VIKING (CMH, Kansas City, MO, USA) was used to display variants characterised by RUNES and, thereby, to interpret WGS findings.^8,34^ VIKING allows input of patient clinical features to sort variants by candidate gene and has additional dynamic filters, including those for minor allele frequency, ACMG variant pathogenicity category, compound heterozygosity and custom gene lists. VIKING enables custom classification of variants, visualisation of read alignments with the IGV and export of analysis findings. On average, genomes contained 825 potentially pathogenic variants (allele frequency <1%, ACMG categories 1–3). All inheritance patterns were examined. Where a single likely causative variant for a recessive disorder was identified, the locus was manually inspected using IGV in the trio for uncalled variants.^58^ Expert interpretation and literature curation were performed for likely causative variants with regard to evidence for pathogenicity.^43^ While rapid WGS can give a provisional diagnosis of genetic disorders in 50 h,^8^ it is a research test, and Sanger sequencing, qPCR, or long-range PCR were used for confirmation of all likely causative genotypes. During the study, the FDA granted ‘non-significant risk’ status to verbal return of a provisional WGS diagnosis to the treating physician in exceptional cases, where the results were actionable and the infant was imminently likely to die (FDA/CDRH/OIR submission Q140271, 8 May 2014). Familial relationships were confirmed by segregation analysis of private variants in WGS diagnoses associated with *de novo* mutations. An infant was classified as having a definitive diagnosis if a pathogenic or likely pathogenic genotype using ACMG criteria in a disease gene that overlapped with a reported phenotype was reported in the medical record.^52^ Expert consultation and functional confirmation were performed when the subject’s phenotype differed from the expected phenotype for that disease gene or if identification of novel disease gene.^59–62^

## Figures and Tables

**Table 1:** Software tools evaluated for performance in detection of DSVs in WGS

*Software tool*	*Primary SV detection methods employed*	*Chr 1 simulation*	*WGS simulation*
Breakdancer	PEM	PASS	PASS
Clever	Read alignment graph and max cliques	PASS	
Cn.MOPS	DOC and Poisson distribution		
Control-freec	SNP B allele frequencies and DOC	PASS	
Dindel	Realignment with probabilistic indel calls		
ERDS	DOC and paired Hidden Markov Model	PASS	
GasvPRO	DOC, PEM and probabilistic model		
GenomeStrip	DOC, PEM and SRM	PASS	PASS
Lumpy	PEM and DOC (SRM with special aligner)	PASS	
SVDetect	DOC, PEM		

Abbreviations: BD, breakdancer; DOC, depth of coverage; ERDS, estimation by read depth with single-nucleotide variants; GS, GenomeStrip; PEM, paired end mapping; SNP, single nucleotide polymorphism; SRM, split read mapping; TP, true positive; WGS, whole-genome sequence.

**Table 2:** Wide differences in the performance of the five SV detection tools that detected a true positive in a simulated Chr 1 DSV data set

*Software tool*	*TP*^a^	*FP*	*FN*	*Recall (sensitivity)*^b^	*Precision (positive predictive value)*^c^	*F2 measure*
Breakdancer	102	24	168	37.78%	81.0%	42.3%
Clever	32	1,683	238	11.9%	1.9%	5.7%
Control-freec	5	449	265	1.9%	1.1%	1.6%
ERDS	149	1,204	121	55.2%	11.0%	30.6%
GenomeStrip	146	673	124	54.1%	17.8%	38.4%
Lumpy	247	526,524	23	91.5%	0.05%	0.2%

Abbreviations: DSV, deletion structural variant; ERDS, estimation by read depth with single-nucleotide variants; FN, false negative; FP, false positive; TP, true positive.

a DSV predictions that overlapped a DSV by >1 nt.

b TP/(TP+FN).

c TP/(TP+FP).

**Table 3:** Performance of the random forest classifier on NA12878 replicates

*NA12878 WGS replicate*	*True positives*	*False positives*	*False negatives*	*Precision*	*Recall*	*F2 measure*
1, BD U GS unfiltered	1,780	2,861	7,958	0.38	0.18	0.31
1, Random forest filtered	1,262	292	8,635	0.81	0.13	0.39
2, BD U GS unfiltered	2,409	4,212	7,525	0.36	0.24	0.33
2, Random forest filtered	1,743	405	8,133	0.81	0.18	0.47
3, BD U GS unfiltered	3,808	18,272	4,553	0.17	0.46	0.20
3, Random forest filtered	3,198	2,867	5,161	0.53	0.38	0.49
4, BD U GS unfiltered	3,521	3,852	5,452	0.48	0.39	0.46
4, Random forest filtered	3,400	67	5,535	0.98	0.38	0.75
Average, BD U GS unfiltered	2,880	7,299	6,372	0.35	0.32	0.32
Average, filtered	2,401	908	6,866	0.78	0.27	0.52

Abbreviations: BD, breakdancer; GS, GenomeStrip; WGS, whole-genome sequence.

**Table 4:** Overlap in BD and GS DSV predictions in three sets of WGS and samples U173 and pg96, showing that filtering increased the r1∩^50%^r2∩^50%^r3 proportion

*Replicates calling DSV*	*Number of BD U GS DSV*			
	*U173*	*U173 filtered*	*pg96*	*pg96 filtered*
Run 1	10,139	1,384	6,340	1,298
Run 2	11,335	1,542	24,033	2,459
Run 3	6,813	988	2,581	1,587
r1∩^50%^r2^a^	12,433	537	2,664	1,674
r2∩^50%^r3	444	341	784	193
r1∩^50%^r3	1,097	1,154	925	544
r1∩^50%^r2∩^50%^r3	237,733	94,335	8,259	3,468
Total DSV calls	279,994	100,281	45,586	11,223
r1∩^50%^r2∩^50%^r3 as % of total	85%	94%	18%	31%

Abbreviations: BD, breakdancer; DSV, deletion structural variant; GS, GenomeStrip; WGS, whole-genome sequence.

a r1∩^50%^r2: DSVs called by BD and GS in run 1 and run 2 with >50% overlap in chromosomal coordinates.
