# Supplementary figures and images for: Clinical detection of deletion structural variants in whole-genome sequences
Source: NPJ Genom Med. 2016 Aug 3;1:16026–. doi: 10.1038/npjgenmed.2016.26 (PMC5685307; doi:10.1038/npjgenmed.2016.26)

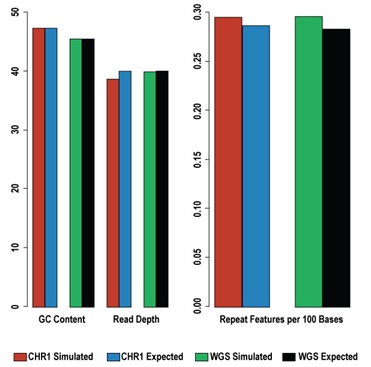

Supplement: Supplementary Figure S1 [file npjgenmed201626-s1.jpg]

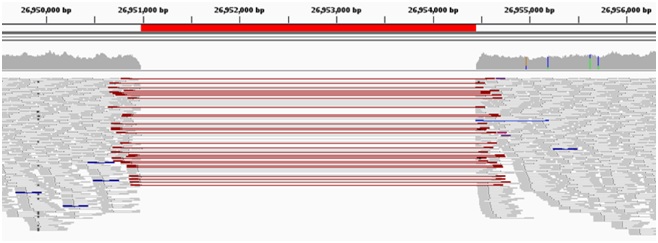

Supplement: Supplementary Figure S2 [file npjgenmed201626-s2.jpg]

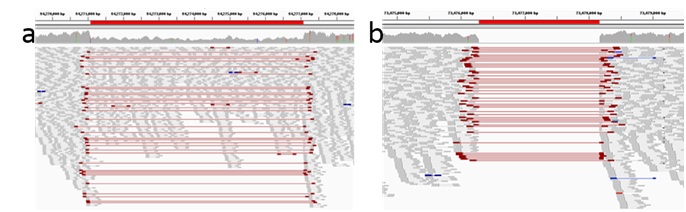

Supplement: Supplementary Figure S3 [file npjgenmed201626-s3.jpg]

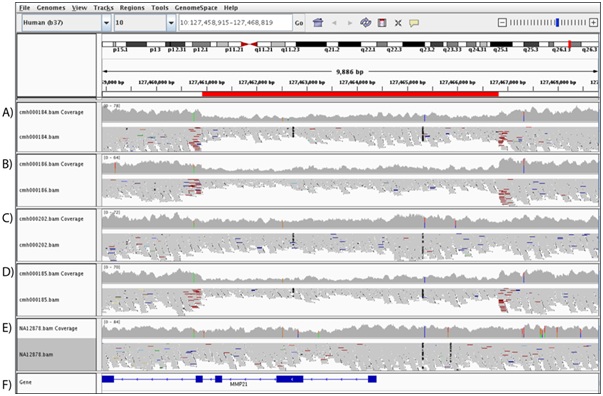

Supplement: Supplementary Figure S4 [file npjgenmed201626-s4.jpg]

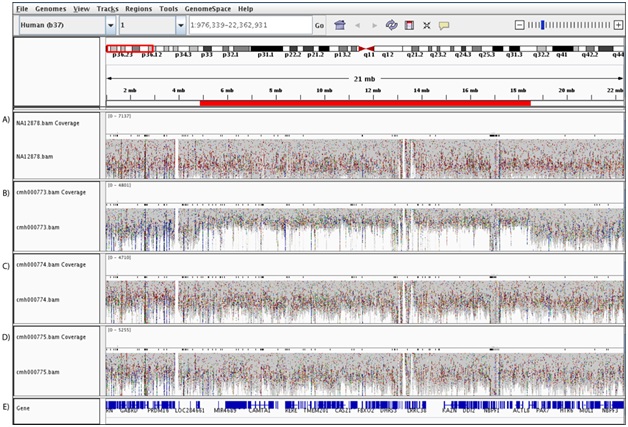

Supplement: Supplementary Figure S5 [file npjgenmed201626-s5.jpg]
